# The Effects of a Short-Term Supplemental Breathwork Protocol on the Aerobic Performance of Recreational Runners

**DOI:** 10.3390/sports13020031

**Published:** 2025-01-23

**Authors:** Adrian T. Wolff, Sara R. Sherman, Craig A. Horswill

**Affiliations:** 1 Department of Kinesiology and Nutrition Sciences, University of Nevada, Las Vegas, NV 89154, USA; 2 College of Applied Health Sciences, University of Illinois Chicago, Chicago, IL 60607, USA; ssherm5@uic.edu; 3 Emeritus, Department of Kinesiology & Nutrition, University of Illinois Chicago, Chicago, IL 60607, USA; horswill@uic.edu

**Keywords:** functional breathing, breathing exercise, breathing pattern, nasal breathing, aerobic training, VO_2max_

## Abstract

We investigated the effects of a functional breathing program on the aerobic performance of recreational runners. Runners participated in an aerobic endurance training program with functional breathing (FBP; *n* = 8, 34.8 ± 5.1 yrs, 25.3 ± 2.5 kg·m^2^) or without functional breathing (CON; *n* = 8, 29 ± 5 yrs, 23 ± 2 kg·m^2^). The treatment group underwent daily breathing exercises, and nasal-only breathing during low-intensity sessions of the training program. The primary outcome variables measured before and after the program included the following: the breath-hold time at rest, the duration and VO_2max_ with nasal-only breathing, and the VO_2max_ with normal breathing during a graded running test. The data were analyzed using two-way ANOVA (*p* < 0.05). We found a significant group x time interaction for breath-hold time (∆ from PRE: +1.9 s [CON], +11.7 s [FBP]; *p* = 0.04; d = 1.13). However, the changes in the time and VO_2max_ with nasal-only breathing, and in the VO_2max_ with normal breathing, did not differ between the FBP and CON groups. A small but significant time (main) effect for the increase in VO_2max_ (~3.0%, *p* < 0.05) suggested that both groups had adequate stimuli for physiological adaptations. The four-week supplementary functional breathing protocol increased the breath-hold time, but not the maximum nasal-only breathing time, nasal-only breathing VO_2max_, or VO_2max_, in recreational runners.

## 1. Introduction

Nasal-only breathing and hypoventilation breathing exercises, used to induce mild to moderate hypoxia at rest and during physical activity, have become popular in recent years for the purposes of relaxation, wellbeing, and possibly improved physical performance [1,2]. Nasal breathing offers the benefits of filtering, warming, and humidifying air prior to it entering the lungs. However, nasal breathing may also reduce the volume of O_2_ inhaled compared to mouth breathing [3]. Prior literature examining the acute effects of nasal-only breathing on aerobic performance demonstrated that maximal oxygen consumption (VO_2max_) decreased threefold compared to VO_2max_ when oral breathing was allowed [4]. At VO_2max_, nasal-only breathing resulted in a lower tidal volume, respiration rate, and fraction of expired oxygen (FeO_2_), with a concomitant increase in the fraction of expired carbon dioxide (FeCO_2_) [4].

Despite these acute effects, restricted air intake might serve as a form of hypoventilation and inspiratory muscle challenge that provokes a change in chemosensitivity (i.e., a greater tolerance for CO_2_ accumulation and an increase in pH), with a reduced necessity to accelerate breathing, within the cardiovascular and neurological systems [5,6,7]. Restrictive breathing techniques for the inspiratory muscles have been shown to improve cardiovascular wellness in older individuals. Inspiratory muscle strength training for 6 weeks reduced blood pressure, improved endothelial function, and reduced reactive oxygen species in adults of 50 to 79 years of age with pre-hypertension [8]. A change in exercise tolerance was not reported in this study. Prior to this, it was demonstrated that inspiratory muscle training in young adults may reduce the metaboreflex, possibly through changing the chemosensitivity of the inspiratory muscles [9]. As the authors of that study and others explain, this adaptation may allow blood flow to be diverted from the inspiratory muscles to the locomotor muscles, and thereby support improved exercise performance [9,10]. The influence of the degree and form of resistance administered on this effect is not clear. Potentially, nasal-only breathing during exercise training might impose a similar challenge to effect performance, but this has remained untested up to this point.

Nasal-only breathing and functional breathing programs (FBPs) (i.e., breath protocols used to optimize the recruitment of the diaphragm or stimulate hypoxia) have been implemented during aerobic training [9,11]. If nasal-only breathing and FBPs limit oxygen delivery to working muscles, they might create an acute stimulus for physiological adaptations like those seen with high-intensity interval training [12], but at lower exercise intensities, including exercises completed at rest. Although limited research exists, chronic adaptations of training using nasal-only breathing have included lower ratings of perceived exertion (RPEs) at various running intensities, greater oxygen extraction from the systemic circulation to the working muscles during exercise, and an ability to maintain a similar running velocity at VO_2max_ compared to oral breathing, despite reduced ventilation [6,7]. Dallam et al. [6] examined nasal vs. oral breathing during a VO_2max_ running test in participants who had trained (self-selected) for six months using nasal-only breathing at various intensities. The authors found no differences in VO_2max_ or time to exhaustion between the nasal-only and oral-only breathing conditions, and no greater increase in blood lactate in the nasal-only condition. Interestingly, nasal-only breathing was able to match the oral-only breathing work levels, with no significant difference in aerobic energy contribution, leading to speculation of an improved running economy and downregulation of the chemoreceptor response to the increased flux of CO_2_ in nasal-only breathing conditions [6]. A popular belief among lay audiences is that hypoventilation breathing exercises at rest may provide increased tolerance for CO_2_ accumulation during exercise [1]; however, this premise currently lacks any scientific evidence.

To date, a well-controlled study with pre- and post-treatment testing with a control group has yet to be conducted to determine whether an FBP that utilizes a combination of nasal-only breathing during running and hypoventilation exercises during rest can enhance aerobic capacity in recreational runners. Therefore, the purpose of the present study is twofold: (1) to determine whether a four-week supplemental FBP is effective in improving maximal running time using nasal-only breathing (MNRT) and maximal nasal-only breathing oxygen consumption (MNBVO_2_) compared to a control group using the same running protocol, but without the FBP; and (2) to determine if there is a relationship between participants’ change in Body Oxygen Level Test (BOLT) time (i.e., breath hold time) and their respective change in MNBVO_2_ (pre- to post-training). We hypothesized that the FBP group would show a significantly larger increase in MNBVO_2_ and MNRT from pre- to post-training, and that the change in BOLT time would show a positive relationship with the change in MNBVO_2_.

## 2. Materials and Methods

### 2.1. Participants

Participants were recruited from local gyms and running clubs, through word of mouth, and using flyers posted in the community. They were healthy, free of cardiovascular disease, did not take any medication for cardiac issues, and could run at least five kilometers comfortably. Their descriptive statistics are reported in Table 1. Each participant provided written informed consent. The study protocol was approved by the University’s Office of Protection of Research Subjects (Protocol 2019-0303).

### 2.2. Study Design

Using stratification for biological sex, participants were randomly allocated to one of two conditions: running without the FBP (CON) vs. running with the FBP (FBP). The randomization was conducted using the Microsoft Excel randomization feature by placing an equal number of males and females into each group (within cells), then randomly assigning each to either CON or FBP. The participants were individually enrolled and tested, so communication between the control and treatment groups did not occur. Assessments of performance were conducted before and after the four-week treatment period.

### 2.3. Performance Assessments

Participants reported to the lab having completed no strenuous physical activity during the 48 h prior. Their resting heart rate was obtained after the participants had been seated for at least five minutes. The BOLT was performed to identify the duration for which participants could comfortably hold their breath after a normal exhale (an indirect measure of chemosensitivity). Participants took at least three normal breaths prior to a normal exhalation to start the breath hold. The BOLT score was calculated as the time (s) from the beginning of breath hold, when the nose was pinched shut, until the termination of the breath hold, when the nose was released. Following the BOLT, and after taking height and body mass measurements, participants secured mouth tape (SomniFix International LLC, Chevy Chase, MD, USA) over their closed lips. Participants were then fitted for a facemask (COSMED, Rome, Italy) and heart rate monitor (H10, Polar Electro Inc., Bethpage, NY, USA), and began the treadmill graded exercise test (GXT). Expired gasses were analyzed using a metabolic cart (TrueOne 2400, ParvoMedics, Park City, UT, USA).

The GXT used was similar to that used in prior research [13]. Participants began running at 4 mph, at 1% grade, for three minutes. The treadmill speed was then increased to 5 mph for the first stage, and incrementally increased by 1 mph for each subsequent three-minute stage, which allowed for the acquisition of steady-state data on heart rate, ventilation, and RPE during the final minute. The stages progressed until the participants reached a time and speed at which they indicated the need to switch to oral breathing (i.e., MNRT). The test was then paused temporarily as the participants straddled the treadmill. The mouth tape was promptly removed to allow normal breathing and the facemask was repositioned securely. Participants resumed running at the same speed at which the test was paused, and metabolic data collection resumed for completion of the test.

Subjects completed the remaining time for the stage in which this pause occurred, and all subsequent stages lasted only two minutes until they reached volitional fatigue. If volitional fatigue was not reached by the end of the fifth stage, the treadmill grade was increased by 2.5% at every stage until volitional fatigue was reached. The main outcome variables included MNBVO_2_ (mL·kg^−1^·min^−1^), defined as the greatest 15 s VO_2_ value prior to MNRT (just before the test pause), and VO_2max_ (mL·kg^−1^·min^−1^), defined as the greatest 15 s VO_2_ value during the GXT. Running economy (mL·kg^−1^·km^−1^) was measured by analyzing the steady-state oxygen consumption for the running speeds during each of the last three stages prior to MNRT (using the average VO_2_ values of the final 15 s of each stage), and was determined by the slope of the line of best fit for the three stages [14,15].

### 2.4. Treatment

Both groups completed the running program on their own. Compliance was ensured by having participants complete a weekly record that was returned to the researchers after the program. The running program was a polarized design, consisting of two training intensities (i.e., high and low) [16]. Training intensities during the program were set using the heart rate derived at the aerobic threshold (AeT) and estimated ventilatory threshold (VT). The AeT was determined using the Maffetone Formula: 180 − Age [17,18], and was used to set the upper limit of low intensity. The VT was determined as the heart rate derived upon visual approximation of where the minute ventilation increased exponentially during the GXT, and was used to set the lower limit of high intensity. Three of the training days were completed at an intensity below the AeT, and a fourth training day was completed above the VT. The total running time per day was matched for both groups.

The FBP group supplemented the program with daily functional breathing exercises, while the CON group did not. FBP participants with BOLT times <30 s were given breathing exercises designed to reduce tidal volume and stimulate a mild hypercapnic response for a short time per session [1]. The mild hypercapnic response (85–94% SpO_2_) was monitored by participants using a fingertip pulse oximeter (Zacurate Pro Series 500DL FBP, Stafford, TX, USA). Participants with higher BOLT times (≥30 s) were given breathing exercises to stimulate the same effects, but for a longer period per session [1]. These breathing exercises were completed on an individual basis, and were not supervised, due to the high number of sessions that needed to be completed (up to six per day). Participants recorded BOLT times on their compliance sheets every day during the four-week program, and submitted them on day seven of each week. The FBP participants were required to attend a familiarization session, during which they were taught the supplemental functional breathing exercises they would use during the running program. The CON participants were familiarized with the four-week running program after completing the pre-test. Participants were not told which specific “group” they were in, or that groups existed. All they were instructed to do was to complete their exercise program as stated. All participants began their respective four-week programs after completing the pre-treatment graded exercise test (pre-GXT), and reported back to the same lab during the sixth week for their post-treatment GXT (post-GXT).

### 2.5. Statistical Analysis

The descriptive data are presented as the mean ± standard deviation (M ± SD). Two-way (group x time) repeated-measures ANOVA was used for hypothesis testing of the outcome variables: MNRT (s), MNBVO_2_ (mL·kg^−1^·min^−1^), VO_2max_ (mL·kg^−1^·min^−1^), running economy (mL·kg^−1^·km^−1^), and BOLT (s). Greenhouse–Geisser correction was applied when sphericity of the data was not met. Bonferroni correction for multiple comparisons was used in the case of significant ANOVA interactions. The Pearson correlation coefficient was used to determine the relationship between ΔBOLT and ΔMNBVO_2_ for the FBP and CON groups, from pre- to post-training. A priori α was set to *p* < 0.05. Effect sizes for the differences between the two groups were calculated with Cohen’s d, using the means and standard deviations for the changes in their respective pre- and post-assessment outcomes [19]. The following general accepted categories were applied: small effect, d < 0.2; moderate effect, d = 0.5; large effect d > 0.8 [19].

## 3. Results

Sixteen participants completed all phases of the study. With the exception of age, no differences existed between the groups in terms of baseline physical characteristics. The mean age for the FBP group was 34.9 + 5.2 y vs. 28.9 + 5.4 y (*p* = 0.02). For the main outcome variables, no differences existed between the groups at baseline; this suggests that the recruitment and randomization was effective for forming the two cohorts. The average completion rate of the running sessions was 99.3% for the CON group and 96.9% for the FBP group. The average completion rate of the breathing sessions for the FBP group was 91.6%. These data were determined using self-completed compliance sheets that were distributed to participants prior to the program.

There were no significant group x time interactions for mean body mass index (BMI), VO_2max_, MNBVO_2_, MNRT, or RE (*p* > 0.05, Table 2). The effect sizes for the differences between each group pre- and post-change were as follows: BMI, d = 1.1; VO_2max_, d = 0.11; MNBVO_2_, d = 0.05; MNRT, d = 0.17; and RE, d = 0.40.

**Figure 1:**
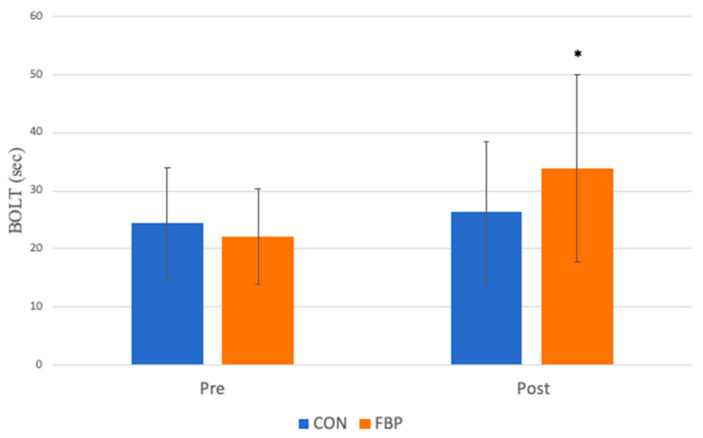
The difference in mean BOLT times between the groups, before and after treatment. There was a significant interaction, such that mean BOLT scores increased for FBP group compared to CON group (*p* < 0.05). * denotes this significant interaction.

**Figure 2:**
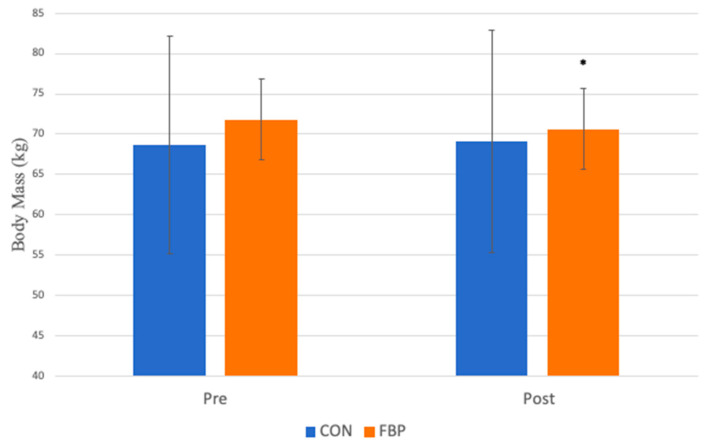
The mean body mass for the groups before and after treatment. An interaction was observed (*p* < 0.01), such that the FBP group lost weight on average, while the CON group gained weight. * denotes this significant interaction.

A significant group x time interaction was observed for the BOLT time (*p* = 0.04; d = 1.13) such that the BOLT time increased significantly more for the FBP group compared to the CON group (Figure 1). There was also an unexpected group x time interaction for body mass (*p* < 0.01; d = 1.78), with the mean body mass decreasing for the FBP group by approximately 1.1 kg, compared to a slight increase of 0.4 kg in the CON group by the end of the treatment period (Figure 2).

**Figure 3:**
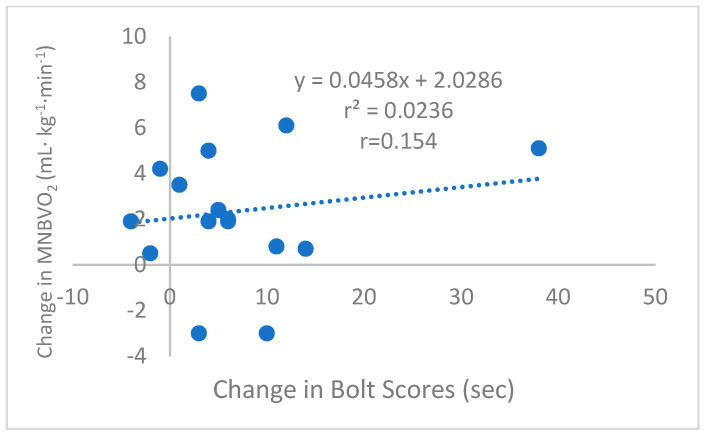
Scatterplot of pre- and post-change scores for BOLT vs. MNBVO_2_, with groups combined (*n* = 16).

While they were not the primary outcomes being tested, significant main effects were noted for the MNRT (*p* = 0.038), MNBVO_2_ (*p* = 0.007), and VO_2max_ (*p* = 0.028). This would suggest that while the treatment period only lasted only 4 weeks, the program was adequate in duration and intensity to stimulate improvements in participants’ aerobic fitness. The relationship between ΔBOLT vs. ΔMNBVO_2_, as seen in Figure 3, was not statistically significant (*r* = 0.15, *p* = 0.570). Likewise, ΔBOLT was not statistically correlated with ΔVO_2max_ or ΔRE.

## 4. Discussion

The purpose of this study was to determine whether a four-week supplemental FBP improved the MNRT and MNBVO_2_ in recreational runners. Furthermore, it sought to examine the relationship between participants’ ΔBOLT and their respective ΔMNBVO_2_. Despite observing an increase in VO_2max_ in both groups, the ΔMNRT and ΔMNBVO_2_ for the FBP group were not different from the control group, and no relationship was found between ΔBOLT and ΔMNBVO_2_. Our rationale for hypothesizing that participants with higher BOLT times would be able to sustain nasal-only breathing at higher submaximal intensities (i.e., a higher MNBVO_2_) stemmed from effects seen from other means of restricting oxygen delivery during exercise [20,21,22,23]. The lack of significant findings for these variables may be attributable to an insufficient program duration and intensity at which the nasal-only breathing was applied. We also tested recreational runners in this study, so it is important to acknowledge that other populations, such as elite runners, may respond differently.

One finding from our study was that the change in ΔBOLT from pre- to post-treatment was significantly higher for the FBP group compared to the CON group (i.e., longer breath-hold time), with a large effect size, suggesting the FBP was effective in increasing breath-hold time. The longer BOLT times in the FBP group may be explained by an adaptation of the body’s chemosensitivity to elevated blood CO_2_ concentrations, and a delayed urge to breathe sooner. Successive attempts of chronic breath holding over the course of four weeks could have resulted in reduced chemosensitivity for the FBP participants. The reduced chemosensitivity could have been caused by habituation to the sensation of dyspnea and elevated blood CO_2_ concentrations [24], like those observed during yogic breathing exercises [25,26] and exposure to a hypercapnic environment for several weeks [27]. Since no significant differences were seen in physical performance-related variables between the treatment and control groups, any changes in chemosensitivity may not extend beyond the resting state to exercise conditions.

Unexpectedly, we observed a reduction in body mass in the FBP group, compared to a slight increase in the CON group. Our study was not designed to address this change, as we did not account for dietary factors, non-exercise activity thermogenesis (NEAT), energy balance, or even the weight-maintenance goals of the subjects. In part, the brevity of the treatment period was meant to focus on the effects of respiratory physiology and avoid longer-term confounding variables such as those that could impact body mass. Therefore, we cannot draw unequivocal conclusions from our data on this specific outcome. However, due to the large effect size, future research may be warranted to determine whether functional breathing exercises per se have an effect on body mass in recreational runners. Interestingly, several studies investigating meditation, mindfulness, and relaxation techniques have reported that breathing exercises may be an effective strategy for weight loss [28,29]. Possibly, the focused attention on breathing and daily respiratory muscle exercises could add to a modest but cumulative daily energy expenditure, much like spontaneous activity, or NEAT [30].

## 5. Limitations

Our sample included males and females, which, in combination, may have added variability to the data. The sample size was also relatively small, due to the preliminary nature of this study. Subjects were asked to complete their training individually, so we do not know whether any unblinding occurred and do not know the degree of compliance, although the records kept by the subjects indicate that compliance was high for the training and breathing exercises. Future research might have subjects report to the lab for supervised training, increase the sample size, and have subjects train for a longer period at higher intensities.

The graded exercise test was unique in that the protocol contained a pause to remove mouth tape and resume oronasal breathing until exhaustion, which made the identification of the VT challenging [13]; however, the method was applied consistently for all subjects, so our comparisons and results should be reliable. Running economy was measured in the stages prior to the respiratory limit being reached, so our calculations may not have reflected true steady-state conditions, even though the stages were 3 min in duration. Future studies might separate the submax testing from the maximum effort testing.

As this was a preliminary study on a novel supplemental functional breathing protocol, we were uncertain of the minimal effective stimulus necessary to elicit physiological adaptations during exercise. The entire training program lasted only four weeks, but it appeared sufficient to increase the VO_2max_ in both groups; this is consistent with previous research showing that VO_2max_ can be significantly increased in four weeks [31], and even in as little as two weeks [32]. In retrospect, the relatively low training intensities may have impacted our results. The prescribed intensities and duration for the nasal-only breathing and breath holds completed at lower intensities may not have been adequate to induce adaptations beyond what was observed in the CON group.

We employed “180 − Age” for the determination of the AeT [17,18]. While this method may be nontraditional in controlled experimental protocols, we chose this formula for its ease of use in enabling participants to maintain a low intensity of effort during runs, and we applied it consistently to ensure reliable results.

The BOLT [1] used as our breath-holding assessment has not been validated in controlled experimental protocols, so it may be prone to variability. However, we attempted to mitigate unreliable breath-holding trials, due to variability between each participant, by providing clear, adequate instruction for consistent performance. Future work might examine nasal-only breathing in moderate- and higher-intensity training, and also consider other outcome variables, such as lactate threshold or onset of blood lactate accumulation (OBLA), as more sensitive indices than VO_2max_ or MNBVO_2_.

## 6. Conclusions

The present study demonstrated that a four-week supplemental FBP in recreational runners did not improve their MNRT, MNBVO_2_, VO_2 max_, or RE at low running intensities compared to the CON group. Participants in the FBP group demonstrated significant improvements in BOLT times compared to the CON group. Future research should consider utilizing nasal-only breathing and breath holds at moderate and high intensities to determine whether recreational runners can increase their MNBVO_2_, MNRT, and anaerobic or lactate threshold under these conditions. The unexpected reduction in body mass for the FBP group might also warrant further investigation.

## Figures and Tables

**Table 1:** Physical characteristics of participants.

Variables	Males (*n* = 8)	Females (*n* = 8)	Combined (*n* = 16)
Age (yr)	33.9 ± 6.3	29.9 ± 5.2	31.9 ± 5.9
Height (m)	1.77 ± 0.1	1.65 ± 0	1.71 ± 0.1
Body Mass (kg)	79.18 ± 7	61.39 ± 7.6	70.28 ± 11.5
BMI (kg·m^2^)	25.3 ± 2.2	22.7 ± 2.7	24 ± 2.7
Pre-Training VO_2max_ (mL·kg^−1^·min^−1^)	50.5 ± 5.7	44.4 ± 6.4	47.5 ± 6.6

Values are reported as mean ± SD.

**Table 2:** Pre- and post-treatment group comparisons.

Variables	CON Pre	CON Post	FBP Pre	FBP Post	*p*-Value
BMI (kg·m^2^)	22.71 ± 2.38	22.82 ± 2.56	25.37 ± 2.55	24.90 ± 2.43	0.075
Body Mass (kg)	68.72 ± 13.49	69.11 ± 13.79	71.83 ± 10	70.66 ± 10.08	0.003 *
VO_2max_ (mL·kg^−1^·min^−1^)	46.83 ± 7.1	47.93 ± 7.44	48.1 ± 6.67	49.52 ± 6.36	0.757
MNBVO_2_ (mL·kg^−1^·min^−1^)	37.46 ± 5.34	39.92 ± 6.14	37.41 ± 3.64	39.63 ± 4.44	0.877
MNRT (s)	707 ± 191.1	778.25 ± 194.7	782 ± 131.6	828.12 ± 145.2	0.631
RE (mL·kg^−1^·km^−1^)	124.15 ± 38.42	146.66 ± 53.43	121.73 ± 49.23	116.09 ± 26.72	0.273
BOLT (s)	24.38 ± 9.6	26.37 ± 12.1	22.13 ± 8.1	33.88 ± 16.0	0.04 *

Values are reported as mean ± SD. * denotes group x time interaction (*p* < 0.05). Running economy (RE).

## Data Availability

Data can be obtained by contacting the corresponding author at the following email address: adrianwolff35@gmail.com.
